# Supplementary material for: YouTube as an information source for bleeding gums: A quantitative and qualitative analysis
Source: PLoS One. 2024 Mar 6;19(3):e0298597. doi: 10.1371/journal.pone.0298597 (PMC10917331; doi:10.1371/journal.pone.0298597)
Supplement: Attachment — Submitted filename: Response to Reviewers.docx [file pone.0298597.s003.docx]

Dear Editor:

We would like to express our sincere gratitude to Dr. Anand Marya for accepting the role of editor for our submitted manuscript titled "YouTube as an information source for bleeding gums: a quantitative and qualitative analysis" (PLOS ONE manuscript number: PONE-D-23-16369). Furthermore, we truly appreciate that you had assigned such qualified reviewers to our manuscript, whose insightful comments and constructive suggestions greatly assisted us in the revision process. We extend our heartfelt thanks for their invaluable contribution. Our point-by-point responses to the comments are provided below:

**Comments from the Editor and Responses:**

1. Concern of the reviewer: Please ensure that your manuscript meets PLOS ONE's style requirements, including those for file naming.

**Our response**: We express our gratitude for your considerate reminder. It is important to note that our article is a research article that adheres to the publication requirements of PLOS ONE. In compliance with the submission guidelines, we have thoroughly reviewed the manuscript, encompassing various aspects such as file format, length, font, title, layout, spacing, page and line numbers, footnotes, language, abbreviations, references, equations, nomenclature, and other pertinent elements. This careful examination was conducted to guarantee that our manuscript aligns with PLOS ONE's style requirements, including the specifications for file naming.

**Revised text**: not applicable.

2. Concern of the reviewer: In your Methods section, please include additional information about your dataset and ensure that you have included a statement specifying whether the collection and analysis method complied with the terms and conditions for the source of the data.

**Our response**: We express our gratitude to the reviewers for drawing our attention to this matter. Additional information regarding our dataset is listed in the supporting information S1 Table, encompassing the video title, video link, video publisher's name, upload source, upload date, days elapsed since upload, expiration date, video length, number of likes, number of views, number of comments, number of subscriptions, viewing rate, usefulness score, Global Quality Score, total score, and DISCERN.

Furthermore, we have included a statement affirming that the methodology employed for data collection and analysis adheres to the guidelines and protocols outlined by both Google Trends and YouTube, this approach has been previously employed in similar studies [1-3]. Google Trends is an internet-based search engine that facilitates the assessment of search phrase frequency within a specific timeframe. It is utilized to identify the most commonly searched keywords of interest [4]. On 28 August 2022 (within a single day), a search on YouTube was conducted using the query "bleeding gums." The analysis demonstrated that the terms "bleeding gums," "teeth bleeding," "bleeding in gums," "stop bleeding gums," and "bleeding gums remedy" were the prevailing expressions employed in connection with this subject matter. By employing Google Trends, it was ascertained that "bleeding gums" was the most frequently queried term. Two researchers conducted a search on the website "https://www.youtube.com" utilizing the identified search terms. According to scholarly investigations, the majority of YouTube viewers tend to explore the top 60 to 200 videos [5]. Consequently, based on these data, the top 200 videos corresponding to the search terms were both viewed and evaluated. This study did not require approval by the local Research Ethics Board as it involved publicly available data only.

**References**

1. Ustdal G, Guney AU. YouTube as a source of information about orthodontic clear aligners. The Angle orthodontist. 2020;90(3):419-424.
2. Sezici YL, Gediz M, Dindaroğlu F. Is YouTube an adequate patient resource about orthodontic retention? A cross-sectional analysis of content and quality. American journal of orthodontics and dentofacial orthopedics. 2022;161(1):e72-e79.
3. Lena Y, Dindaroğlu F. Lingual orthodontic treatment: A YouTube™ video analysis. The Angle orthodontist. 2018;88(2):208-214.
4. Google Trends[Internet]. America: Google; c2022[cited 2022 Aug 28]. Available from: <https://trends.google.com.tr/trends/?geo=TR.>
5. Desai T, Shariff A, Dhingra V, Minhas D, Eure M, Kats M. Is content really king? An objective analysis of the public's response to medical videos on YouTube. PLoS One. 2013;8(12):e82469.

**Revised text**:

**Reviewer Comments and Responses:**

**Reviewer 1:**

1. Concern of the reviewer: Is the manuscript technically sound, and do the data support the conclusions?

**Our response**: We express our gratitude to the reviewers for drawing our attention to this matter. In this study, the evaluation of the included videos involved the integration of The Global Quality Score [1,2] and the usefulness score [3], while the reliability of the videos was assessed using DISCERN [4].

The Global Quality Score (GQS), a five-component scale used to estimate the educational value of each video, was utilized to assess the quality of all selected videos [5].

Since the video was primarily intended for non-professional audiences, the five main contents of the video were evaluated systematically based on the usefulness score [3], which included local and systemic etiologies, symptoms, treatment, and prognosis. The combination of the Global Quality Score and the usefulness score determines the total score.

To assess the dependability of YouTube videos, a modified version of the DISCERN tool developed by Charnock et al. [4] was employed. The modified DISCERN reliability tool consisted of five questions, each of which required a yes or no response [4,6]. This was a five-item questionnaire (score: 1 to 5) to evaluate health information. Each "yes" response was worth 1 point; the maximum score was 5 [4,6]. A score of 5 denoted excellently, 4 indicated good, 3 indicated moderate, 2 generally indicated generally poor reliability and a score of 1 indicated poor reliability [7].

In previous research conducted by Kaval et al. and Ustdal et al. [2,8], the GQS and DISCERN assessment tools were employed to evaluate the videos included in their studies. Their findings indicated significant associations between video length, GQS, and DISCERN. Additionally, Capan et al. [3] utilized the usefulness score as a metric to evaluate the videos included in their investigation, revealing a positive correlation between video duration and the usefulness score.

In the present study, the average duration of the video, number of likes, and number of comments on "poor," "medium," and "good" videos were substantially different (p <0.05); the number of likes, viewership, usefulness score, GQS, and DISCERN were all positively connected with video duration (p <0.05); videos longer than 4 minutes in length had higher average DISCERN, usefulness score, and GQS. The result shows that video length was moderately correlated with GQS, usefulness score, and DISCERN, consistent with the studies of Kaval, Ustdal, and Capan, et al. [2,3,8]

**References**

1. Sezici YL, Gediz M, Dindaroğlu F. Is YouTube an adequate patient resource about orthodontic retention? A cross-sectional analysis of content and quality. American journal of orthodontics and dentofacial orthopedics. 2022;161(1):e72-e79.
2. Ustdal G, Guney AU. YouTube as a source of information about orthodontic clear aligners. The Angle orthodontist. 2020;90(3):419-424.
3. Çapan BŞ. YouTube as a source of information on space maintainers for parents and patients. PLoS One. 2021;16(2):e0246431.
4. Charnock D, Shepperd S, Needham G, Gann R. DISCERN: an instrument for judging the quality of written consumer health information on treatment choices. Journal of Epidemiology and Community Health. 1999;53(2):105-11.
5. Bernard A, Langille M, Hughes S, Rose C, Leddin D, Veldhuyzen van Zanten S. A systematic review of patient inflammatory bowel disease information resources on the World Wide Web. The American journal of gastroenterology. 2007;102(9):2070-2077.
6. Ng JY, Ahmed S, Zhang CJ. Dietary and herbal supplements for weight loss: Assessing the quality of patient information online. Nutrition Journal. 2021;20:1-13.
7. Aksoy M, Topsakal KG. YouTube™ for information on paediatric oral health instructions. International Journal of Dental Hygiene. 2022;20(3):496-503.
8. Kaval ME, Demirci GK, Atesci AA, Sarsar F, Dindaroğlu F, Güneri P, et al. YouTube™ as an information source for regenerative endodontic treatment procedures: Quality and content analysis. International Journal of Medical Informatics. 2022;161:104732.

**Revised text**: not applicable.

2. Concern of the reviewer: Has the statistical analysis been performed appropriately and rigorously?

**Our response**: We express our gratitude for your considerate reminder. We understand the significance of rigorous statistical analysis. Therefore, prior to commencing our study, we consulted a statistical expert and conducted a review of relevant literature [1-4]. Aligning with the surveys conducted by Lena, Kaval, Sezici, Fortuna, et al. [1-4], statistical tests were performed using IBM SPSS Statistics 27 software. The Shapiro-Wilk test was utilized to assess data normality, while the Kruskal-Wallis test was employed to compare video characteristics among videos categorized as 'poor', 'moderate', and 'good', the Mann-Whitney test was conducted to identify disparities between video variable characteristics and video length. Additionally, Spearman's correlation coefficient was utilized to evaluate associations among general YouTube video characteristics.

**References**

1. Lena Y, Dindaroğlu F. Lingual orthodontic treatment: A YouTube™ video analysis. The Angle orthodontist. 2018;88(2):208-214.
2. Kaval ME, Demirci GK, Atesci AA, Sarsar F, Dindaroğlu F, Güneri P, et al. YouTube™ as an information source for regenerative endodontic treatment procedures: Quality and content analysis. International Journal of Medical Informatics. 2022;161:104732.
3. Sezici YL, Gediz M, Dindaroğlu F. Is YouTube an adequate patient resource about orthodontic retention? A cross-sectional analysis of content and quality. American journal of orthodontics and dentofacial orthopedics. 2022;161(1):e72-e79.
4. Fortuna G, Schiavo JH, Aria M, Mignogna MD, Klasser GD. The usefulness of YouTube™ videos as a source of information on burning mouth syndrome. Journal of oral rehabilitation. 2019;46(7):657-665.

**Revised text**: We thank Dr. Weijun Zheng (Department of Medical Statistics, Zhejiang Chinese Medicine University, China) for providing professional instructions in statistical analyses.

3. Concern of the reviewer: Is the manuscript presented in an intelligible fashion and written in standard English?

**Our response**: We express our gratitude for your considerate reminder. We have invited a professional editorial team(Freescience Editorial Team) to assist us in polishing articles so that they are intelligible and written in proper English. Furthermore, we aspire for the revised manuscript to meet your acceptance.

**Revised text**: not applicable.

4. Concern of the reviewer: What is novelty of your research ??

**Our response**:

We express our gratitude to the reviewers for drawing our attention to this matter, the innovations of our research are listed below:

1. Bleeding gums present a prevalent dental issue in individuals' everyday lives [1-3]. The heightened risk of COVID-19 infection arises from the close proximity between dental practitioners and patients, as well as the generation of aerosols within dental settings [4-6]. Consequently, individuals have refrained from visiting dental clinics amidst the COVID-19 pandemic [4]. In this context, the utilization of the internet can effectively mitigate transmission rates by minimizing physical contact, particularly during the current outbreak. The utilization of the YouTube platform for the purpose of seeking health-related information has become prevalent among individuals, primarily attributed to the heightened prominence of social networks and the Internet, as well as the convenience and accessibility of the information accessible through this medium. Notably, this inclination towards employing such applications has witnessed a notable surge amidst the ongoing COVID-19 pandemic [7].
2. YouTube, a widely utilized video-sharing platform [8], garners immense popularity with over 2 billion daily views [9] and serves as a prominent destination for individuals seeking medical knowledge. It is important to note that YouTube operates on an open-access model, wherein user-generated content lacks the scrutiny of peer review. While YouTube effectively provides valuable health education resources, it is imperative to acknowledge the potential prevalence of misinformation and inaccuracies [9]. Dental professionals have acknowledged the significance of YouTube as a source of patient information, consequently, numerous studies have evaluated the informational quality of YouTube videos on oral-related topics such as burning mouth syndrome, root canal treatment, lingual orthodontic treatment, regenerative endodontic treatment et al. [10-13]
3. According to our knowledge, no recent study has looked into bleeding gums-related content on YouTube. Incorrect and inadequate content in YouTube videos about bleeding gums can significantly influence patients' attitudes and medical decisions. Consequently, the objective of this study is to perform both quantitative and qualitative analyses of YouTube videos pertaining to bleeding gums. In line with the investigations conducted by Atik, Giulio, et al. [10,14] on Halitosis and Burning Mouth Syndrome, the quality of videos was poor, Atik et al. 's study [14] is explained in our discussion section. While one might expect videos related to bleeding gums to similarly show poor quality, our research shows that such videos actually show a moderate level of quality, our results are different from theirs [10,14].

**References**

1. Lawal FB, Dosumu EB. Self-reported and clinically evident gingival bleeding and impact on oral health-related quality of life in young adolescents: a comparative study. Malawi medical journal. 2021;33(2):121-126.
2. Molina GF, Faulks D, Mazzola I, Cabral RJ, Mulder J, Frencken JE. Three-year survival of ART high-viscosity glass-ionomer and resin composite restorations in people with disability. Clinical oral investigations. 2018;22(1):461-467.
3. Chen H, Zhang R, Cheng R, Xu T, Zhang T, Hong X, et al. Gingival bleeding and calculus among 12-year-old Chinese adolescents: a multilevel analysis. BMC Oral Health. 2020;20(1):147.
4. Loch C, Kuan IB, Elsalem L, Schwass D, Brunton PA, Jum'ah A. COVID‐19 and dental clinical practice: Students and clinical staff perceptions of health risks and educational impact. Journal of Dental Education. 2021;85(1):44-52.
5. Haridy R, Abdalla MA, Kaisarly D, Gezawi ME. A cross‐sectional multicenter survey on the future of dental education in the era of COVID‐19: Alternatives and implications. Journal of Dental Education. 2021;85(4):483-93.
6. Iyer P, Aziz K, Ojcius DM. Impact of COVID‐19 on dental education in the United States. Journal of Dental Education. 2020;84(6):718-22.
7. Aksoy M, Topsakal KG. YouTube™ for information on paediatric oral health instructions. International Journal of Dental Hygiene. 2022;20(3):496-503.
8. Bezner SK, Hodgman EI, Diesen DL, Clayton JT, Minkes RK, Langer JC, et al. Pediatric surgery on YouTube™: is the truth out there? Journal of Pediatric Surgery. 2014;49(4):586-9.
9. Hassona Y, Taimeh D, Marahleh A, Scully C. YouTube as a source of information on mouth (oral) cancer. Oral Diseases. 2016;22(3):202-8.
10. Fortuna G, Schiavo JH, Aria M, Mignogna MD, Klasser GD. The usefulness of YouTube™ videos as a source of information on burning mouth syndrome. Journal of oral rehabilitation. 2019;46(7):657-665.
11. Nason K, Donnelly A, Duncan HF. YouTube as a patient-information source for root canal treatment. International endodontic journal. 2016;49(12):1194-1200.
12. Lena Y, Dindaroğlu F. Lingual orthodontic treatment: A YouTube™ video analysis. The Angle orthodontist. 2018;88(2):208-214.
13. Kaval ME, Demirci GK, Atesci AA, Sarsar F, Dindaroğlu F, Güneri P, et al. YouTube™ as an information source for regenerative endodontic treatment procedures: Quality and content analysis. International Journal of Medical Informatics. 2022;161:104732.
14. Ramadhani A, Zettira Z, Rachmawati YL, Hariyani N, Maharani DA. Quality and Reliability of Halitosis Videos on YouTube as a Source of Information. Dent J (Basel). 2021;9(10):120.

**Revised text**: not applicable.

5. Concern of the reviewer:the methodology seems vague particularly study design??Clarify it.

**Our response**: We appreciate this comment from the reviewer and thank them for highlighting this very valid point. The comprehensive depiction of the present research design can be found in the search strategy, as the initial portrayal of the study design section may potentially perplex readers. In light of this, we have made appropriate revisions, referring to the previous study conducted by Antonio et al [1-3].

**References**

1. Romano A, Lauritano D, Fiori F, Di Petrillo M, Hasan I, Lucchese A, et al. Cross-sectional study on the quality of oral lichen planus videos on YouTube™. Journal of oral pathology & medicine. 2021 Feb;50(2):220-228.
2. Sezici YL, Gediz M, Dindaroğlu F. Is YouTube an adequate patient resource about orthodontic retention? A cross-sectional analysis of content and quality. American journal of orthodontics and dentofacial orthopedics. 2022;161(1):e72-e79.
3. Uzel İ, Ghabchi B, Akalın A, Eden E. YouTube as an information source in paediatric dentistry education: Reliability and quality analysis. PLoS One. 2023;18(3):e0283300.

**Revised text**:

6. Concern of the reviewer: the inclusion and exclusion criteria also needs to be clarified.

**Our response**: We express our gratitude to the reviewers for drawing our attention to this matter. To effectively identify videos pertaining to bleeding gums, it is imperative to establish explicit and attainable inclusion and exclusion criteria [1]. In light of this, we have devised an Inclusion and Exclusion Criteria based on relevant prior research [2-6] and subsequently conducted an initial screening of the videos. Because our original manuscript didn’t include relevant references, it may confuse readers. For this reason, we have added some references using the relevant inclusion and exclusion criteria in the present manuscript.

With reference to Prabhadevi, Nason, and Yagmur et al [2-4], our inclusion criteria were: 1. Video quality of 240p or more. 2. The video was in English (not in any other language such as Chinese, Arabic, and French). 3. The duration of the video was limited to 15 min (longer videos were less likely to capture the attention of online viewers, according to a previous study, so the video was restricted to 15 min [3]).

In accordance with the works of Kübra et al. [5], our exclusion criteria were: 1. Repetition of the video.  2. Videos without audio and description. 3. Inadequate audio quality. 4. Irrelevant to the topic. 5. Deleted or private, therefore unreachable.

After application of the exemption criteria, we excluded 93 (46.5%) videos, and 107 (53.5%) videos were included in the further assessment.

**References**

1. Takhar SS, Schriger DL, Barrett TW. Some think antibiotics are candy, but we know they're not: answers to the May 2012 Journal Club Questions. Annals of emergency medicine. 2012;60(4):528-534.
2. Maganur PC, Hakami Z, Raghunath RG, Vundavalli S, Jeevanandan G, Almugla YM, et al. Reliability of Educational Content Videos in YouTubeTM about Stainless Steel Crowns. Children (Basel). 2022;9(4):571.
3. Nason K, Donnelly A, Duncan HF. YouTube as a patient-information source for root canal treatment. International endodontic journal. 2016;49(12):1194-1200.
4. Sezici YL, Gediz M, Dindaroğlu F. Is YouTube an adequate patient resource about orthodontic retention? A cross-sectional analysis of content and quality. American journal of orthodontics and dentofacial orthopedics. 2022;161(1):e72-e79.
5. Topsakal KG, Duran GS, Görgülü S, Eser Misir S. Is YouTubeTM an adequate source of oral hygiene education for orthodontic patients?. International journal of dental hygiene. 2022;20(3):504-511.

**Revised text**:

7. Concern of the reviewer: How you accessed the video quality?? is there any gold standrad??

**Our response**: We express our gratitude to the reviewers for drawing our attention to this matter. There is no gold standard for evaluating the quality of video information on video-sharing sites, The Global Quality Score and the usefulness score are commonly used quality rating criteria and have also been used in many previous YouTube studies [1-4]. So we used two different rating systems to evaluate the quality of videos about bleeding gums on YouTube. A comprehensive explanation of the methodology for conducting video quality analysis can be found in question 1 above(Is the manuscript technically sound, and does the data support the conclusions? ).

**References**

1. Sezici YL, Gediz M, Dindaroğlu F. Is YouTube an adequate patient resource about orthodontic retention? A cross-sectional analysis of content and quality. American journal of orthodontics and dentofacial orthopedics. 2022;161(1):e72-e79.
2. Ustdal G, Guney AU. YouTube as a source of information about orthodontic clear aligners. The Angle orthodontist. 2020;90(3):419-424.
3. Bernard A, Langille M, Hughes S, Rose C, Leddin D, Veldhuyzen van Zanten S. A systematic review of patient inflammatory bowel disease information resources on the World Wide Web. The American journal of gastroenterology. 2007;102(9):2070-2077.
4. Çapan BŞ. YouTube as a source of information on space maintainers for parents and patients. PLoS One. 2021;16(2):e0246431.

**Revised text**: not applicable.

**Reviewer 2:**

1. Concern of the reviewer: “According to the new classification of periodontal and peri-implant diseases and conditions for 2017.” Please find the latest classification.“

**Our response**: We express our gratitude to the reviewer for their invaluable suggestion. The World Workshop on the Classification of Periodontal and Peri-Implant Diseases and Conditions was held in Chicago from November 9 to 11, 2017. More than 100 participants from all over the world revised the 1999 periodontal disease classification, proposed a new classification framework for periodontitis, and developed the classification of peripheral diseases and conditions for the first time with reference to the periodontal disease classification scheme. It was known that a new classification of periodontal and peri-implant diseases and conditions was summarized and published in June 2018. The relevant content has been appropriately modified in accordance with the new classification.

**Revised text**:

2. Concern of the reviewer: Gingivitis, Periodontitis and peri-implantitis are common diseases. Peri-implantitis also can result in bleeding gums. Please add this.

**Our response**: We express our gratitude for bringing attention to this significant matter and for providing us with the relevant literature [1,2]. We carefully read these two kinds of literature, and by referring to these two kinds of literature [1,2], we have incorporated the information regarding the association between Peri-implantitis and bleeding gums into the manuscript.

According to H.W. et al. [3], there has been a substantial rise in the prevalence of dental implants, with figures increasing from 0.7% in 1999-2000 to 5.7% in 2015-2016. Projections for dental implant prevalence in 2026 range from 5.7% in the most conservative scenario to 23% in the least conservative scenario. These findings demonstrate a considerable increase in the utilization of dental implants for replacing missing teeth since 1999. However, it is important to note that overall access to dental implants remains limited. According to Dinesh, Chun-Teh et al, [1,4] the prevalence rates of implant-based and subject per-implants were determined to be 9.25% and 19.83%, respectively, based on weighted averages. Similarly, the weighted mean prevalence rates of implant-based and subject perimplant-based mucositis were found to be 29.48% and 46.83%, respectively.

However, the Global Burden of Disease Study conducted in 2016 revealed that periodontal disease ranked as the 11th most prevalent condition globally [2,5]. It is worth noting that periodontal disease, encompassing gingivitis and periodontitis, affects a substantial proportion of the global population, with estimates suggesting a prevalence rate of up to 90% [6].

Consequently, it can be inferred that periodontal disease is the primary cause of bleeding gums.

**References**

1. Rokaya D, Srimaneepong V, Wisitrasameewon W, Humagain M, Thunyakitpisal P. Peri-implantitis Update: Risk Indicators, Diagnosis, and Treatment. European journal of dentistry. 2020;14(4):672-682.
2. Heboyan A, Syed AUY, Rokaya D, Cooper PR, Manrikyan M, Markaryan M. Cytomorphometric Analysis of Inflammation Dynamics in the Periodontium Following the Use of Fixed Dental Prostheses. Molecules. 2020;25(20):4650.
3. Elani HW, Starr JR, Da Silva JD, Gallucci GO. Trends in Dental Implant Use in the U.S., 1999-2016, and Projections to 2026. Journal of dental research. 2018;97(13):1424-1430.
4. Lee CT, Huang YW, Zhu L, Weltman R. Prevalences of peri-implantitis and peri-implant mucositis: systematic review and meta-analysis. Journal of dentistry. 2017;62:1-12.
5. Achoki T, Miller-Petrie MK, Glenn SD, Kalra N, Lesego A, Gathecha GK, et al. Health disparities across the counties of Kenya and implications for policy makers, 1990-2016: a systematic analysis for the Global Burden of Disease Study 2016. Lancet Glob Health. 2019 Jan;7(1):e81-e95.
6. Pihlstrom BL, Michalowicz BS, Johnson NW. Periodontal diseases. Lancet. 2005;366(9499):1809-1820.

**Revised text**:

3. Concern of the reviewer:“A YouTube search for "bleeding gums" was conducted on 28 August 2022”. Is the search done only one day or for a specific duration? Please elaborate.

**Our response**: We express our gratitude to the reviewers for drawing our attention to this matter. The search is only for one day, the dynamic nature of YouTube™ algorithms can be considered as the limitation of the present study since the uploaders may delete, modify and edit the videos, comments and descriptive data. The vulnerability of videos to further interventions would eventually lead to variations among the search results in different time periods [1]. Thus, new additions and/or modifications of the videos may be present after 28 August 2022, which is the date of the analyses of the included videos, and would lead to variations among the findings. The manuscript has provided a description of the pertinent limitations.

**References**

1. Kaval ME, Demirci GK, Atesci AA, Sarsar F, Dindaroğlu F, Güneri P, et al. YouTube™ as an information source for regenerative endodontic treatment procedures: Quality and content analysis. International Journal of Medical Informatics. 2022;161:104732.

**Revised text**:

4. Concern of the reviewer: “The duration of the video was limited to 15 min (longer videos were less likely to capture the attention of online viewers, according to a previous study, so the video was restricted to 15 min).”This is not really true. Some patient having bleeding gum if wants to know more may want to know more about it can see or listen the longer and detail videos.

**Our response**:We express our gratitude to the reviewer for their invaluable suggestion.

Length is one aspect of a video that plays an important role in determining the viewer's attention level. Research has found that in learning videos, it is crucial to use shorter lengths to maximize the audience's attention. The longer the video, the less attention the viewer will pay [1]. According to Cynthia's research [2], videos that are less than 6 minutes long are watched more often, and videos that span more than 6 to 9 minutes reduce the audience's attention. According to Luan's research [3], the duration of videos that are beneficial to viewers is about seven minutes. This suggests that the video duration must be long enough to fully cover the content, but the optimal video duration must be considered to avoid losing the viewer's attention.

In a prior investigation conducted by Connor, Nason, and Capan et al [4-6]. pertaining to Femoroacetabular Impingement, root canal treatment, and space maintainers, the researchers restricted the video duration to 15 minutes. Their findings indicated it should be noted that lengthier videos are less likely to attract online viewers.

Hence, in accordance with their research [4-6], we imposed a constraint on the video duration, limiting it to 15 minutes. Our data shows that videos less than 4 min in length have higher average views, which is consistent with the findings of Connor, Nason, and Capan et al. [4-6]

Notably, we excluded certain videos longer than 15 minutes that might have valuable content, a limitation that has been illustrated in the manuscript.

**References**

1. Ramadhani A, Zettira Z, Rachmawati YL, Hariyani N, Maharani DA. Quality and Reliability of Halitosis Videos on YouTube as a Source of Information. Dent J (Basel). 2021;9(10):120.
2. Brame CJ. Effective Educational Videos: Principles and Guidelines for Maximizing Student Learning from Video Content. CBE life sciences education. 2016;15(4):es6.
3. Kovalski LNS, Cardoso FB, D'Avila OP, Corrêa APB, Martins MAT, Martins MD, et al. Is the YouTube™ an useful source of information on oral leukoplakia?. Oral diseases. 2019;25(8):1897-1905.
4. Crutchfield CR, Frank JS, Anderson MJ, Trofa DP, Lynch TS. A Systematic Assessment of YouTube Content on Femoroacetabular Impingement: An Updated Review. Orthopaedic journal of sports medicine. 2021;9(6):23259671211016340.
5. Nason K, Donnelly A, Duncan HF. YouTube as a patient-information source for root canal treatment. International endodontic journal. 2016;49(12):1194-1200.
6. Çapan BŞ. YouTube as a source of information on space maintainers for parents and patients. PLoS One. 2021;16(2):e0246431.

**Revised text**: not applicable.

5. Concern of the reviewer: How many researchers did the search. Needs to add.

**Our response**: We express our gratitude for your considerate reminder, sorry this detail is not explained, in our study, two researchers did the search, and the relevant detail has been added.

**Revised text**:

Thank you for your consideration. I look forward to hearing from you.

Best Regards.

Prof. Minkui Lin

Email address: linmk105@sina.com.
