# Supplementary material for: YouTube as an information source for bleeding gums: A quantitative and qualitative analysis
Source: PLoS One. 2024 Mar 6;19(3):e0298597. doi: 10.1371/journal.pone.0298597 (PMC10917331; doi:10.1371/journal.pone.0298597)
Supplement: Attachment — Submitted filename: Response to Reviewers.docx [file pone.0298597.s004.docx]

Dear Editor:

Thank you for giving us the opportunity to submit a revised version of “YouTube as a Source of Information on Bleeding Gums: A Quantitative and Qualitative Analysis” (PLOS ONE manuscript number: PONE-D-23-16369) for potential publication in PLOS ONE. We appreciate your and the reviewers’ time and effort in providing feedback on our article. Your views, as well as the reviewers’, are valuable and have been instrumental in improving our research papers. We have carefully reviewed all of the suggestions and have made our best efforts to incorporate them into the text in order to meet the acceptance criteria. The following is our peer-to-peer response to these comments:

**Reviewer Comments and Responses:**

1. If the authors have adequately addressed your comments raised in a previous round of review and you feel that this manuscript is now acceptable for publication, you may indicate that here to bypass the “Comments to the Author” section, enter your conflict of interest statement in the “Confidential to Editor” section, and submit your "Accept" recommendation.

Reviewer #1: (No Response)

Reviewer #3: (No Response)

**Our response**: None	.

2. Is the manuscript technically sound, and do the data support the conclusions?

Reviewer #1: Partly

Reviewer #3: Yes

**Our response**: We appreciate the reviewer’s acknowledgement of our efforts to improve the manuscript based on the comments. However, there are still some areas that require clarification.

Firstly, we would like to point out that our choice of viewing and reviewing the top 200 videos was based on a study [1] indicating that the majority of YouTube viewers search within this range. This allowed us to have a suitable sample size for analysis.

Furthermore, the analytical techniques employed in our study are well-established. For instance, we utilized the global quality score (GQS) [2,3], usefulness score [4], and DISCERN [5], all of which have been extensively used in peer-reviewed studies and serve to strengthen and enhance our conclusions.

Finally, we agree with the reviewer that collecting high-quality data is a critical initial step in establishing the validity of the findings. To ensure this, we had two researchers independently examine and rate each video, and the reliability of the raters was assessed before commencing the analysis of the videos. In light of the reviewer’s recommendation regarding the manuscript data, we have re-evaluated our statistical methods and experimental data.

To sum up, we tried our best to study the topic using classical methods from numerous literatures, and have obtained the data and information to reach the conclusions of this manuscript.

**References**

1. Desai T, Shariff A, Dhingra V, et al. Is content really king? An objective analysis of the public's response to medical videos on YouTube. PLoS One. 2013; 8(12): e82469.
2. Sezici YL, Gediz M, Dindaroğlu F. Is YouTube an adequate patient resource about orthodontic retention? A cross-sectional analysis of content and quality. Am J Orthod Dentofacial Orthop. 2022; 161(1): e72-e79.
3. Ustdal G, Guney AU. YouTube as a source of information about orthodontic clear aligners. Angle Orthod. 2020; 90(3): 419-424.
4. Çapan BŞ. YouTube as a source of information on space maintainers for parents and patients. PLoS One. 2021; 16(2): e0246431.
5. Charnock D, Shepperd S, Needham G, et al. DISCERN: an instrument for judging the quality of written consumer health information on treatment choices. J Epidemiol Community Health. 1999; 53(2): 105-111.

**Revised te**xt: not applicable. (The statistical methods and the revised text related are seen in our third response)

3. Has the statistical analysis been performed appropriately and rigorously?

Reviewer #1: I Don't Know

Reviewer #3: Yes

**Our response**: We sincerely apologize for not providing sufficient clarity regarding the appropriateness of our statistical analysis in our previous response letter. Prior to evaluating the videos, we consulted with statistical experts and carefully reviewed pertinent literature [1-4]. To ensure reliability among raters, we calculated Kappa coefficients of correlation. The Shapiro-Wilk test was employed to determine whether our data conformed to a normal distribution. For datasets that deviated from normality, we utilized non-parametric tests: specifically, the Kruskal-Wallis test for comparing more than two samples, the Mann-Whitney test for comparing two independent samples, and Spearman's correlation coefficient to assess relationships between variables.

In our revised manuscript, we have included extensive details on our statistical methodologies, elucidating the specific tests employed and the rationales behind their selection. Furthermore, to enhance transparency and readability, we have clearly indicated which statistical tests were used within each chart legend. This added clarity aims to facilitate a thorough understanding of our analytical approach and the robustness of our findings.

**References**

1.Lena Y, Dindaroğlu F. Lingual orthodontic treatment: A YouTube™ video analysis. Angle Orthod. 2018; 88(2): 208-214.

2.Kaval ME, Demirci GK, Atesci AA, et al. YouTube™ as an information source for regenerative endodontic treatment procedures: Quality and content analysis. Int J Med Inform. 2022; 161: 104732.

3.Sezici YL, Gediz M, Dindaroğlu F. Is YouTube an adequate patient resource about orthodontic retention? A cross-sectional analysis of content and quality. Am J Orthod Dentofacial Orthop. 2022; 161(1): e72-e79.

4.Fortuna G, Schiavo JH, Aria M, et al. The usefulness of YouTube™ videos as a source of information on burning mouth syndrome.J Oral Rehabil. 2019; 46(7): 657-665.

**Revised text**:

4. Have the authors made all data underlying the findings in their manuscript fully available?

Reviewer #1: No

Reviewer #3: Yes

**Our response**: We are immensely grateful for your thoughtful reminder, as we recognize that due to space constraints, it is not always possible to include all relevant information in the main manuscript. We strive to provide any previously omitted information wherever feasible. Unfortunately, the size of the 200 videos in this study, amounting to 2.78GB, exceeded the file upload limit of PLOS ONE, preventing us from uploading them. Therefore, we have included comprehensive details of the 107 included videos in the S1 Table and provided reasons for the exclusion of 93 videos in the S2 Table.

**Revised text**:

5. Is the manuscript presented in an intelligible fashion and written in standard English?

Reviewer #1: No

Reviewer #3: Yes

**Our response**: Thank you for your thoughtful suggestion. Based on your recommendation, we have re-engaged a professional editorial team (AJE editorial team) to assist us in polishing the manuscript. We have thoroughly revised the entire manuscript to ensure that it is easy to understand and written in standard English. We sincerely hope that the revised manuscript will meet your satisfaction.

**Revised text**:

6. Review Comments to the Author

**Reviewer #1:** Dear Author,

The rebuttal against the raised queries are not satisfactory and not addressed as expected.

We have carefully reconsidered the 1st round queries from Reviewer #1 and aim to ensure the following updated responses fully satisfies your needs.

(1) What is novelty of your research ?

**Our response**: We appreciate the opportunity to explain the novelty of our research and to make revisions. We noted that our previous abstract did not highlight the novelty of this paper, so we revised the abstract to highlight what is novel about our study.

In short, bleeding gums present a prevalent dental issue in individuals' everyday lives [1-3]. The heightened risk of COVID-19 infection arises from the close proximity between dental practitioners and patients, as well as the generation of aerosols within dental settings [4-6]. Consequently, individuals have refrained from visiting dental clinics amidst the COVID-19 pandemic [4]. In this context, the utilization of the internet can effectively mitigate transmission rates by minimizing physical contact, The utilization of the YouTube platform for the purpose of seeking health-related information has become prevalent among individuals, and it is important to note that YouTube operates on an open-access model, wherein user-generated content lacks the scrutiny of peer review. While YouTube effectively provides valuable health education resources, it is imperative to acknowledge the potential prevalence of misinformation and inaccuracies [7]. Dental professionals have acknowledged the significance of YouTube as a source of patient information, consequently, numerous studies have evaluated the informational quality of YouTube videos on oral-related topics such as burning mouth syndrome, root canal treatment, lingual orthodontic treatment, regenerative endodontic treatment et al [8-11]. According to our knowledge, no recent study has looked into bleeding gums-related content on YouTube. Incorrect and inadequate content in YouTube videos about bleeding gums can significantly influence patients' attitudes and medical decisions. Consequently, the objective of this study is to perform both quantitative and qualitative analyses of YouTube videos pertaining to bleeding gums.

In our revision, we have directly stated the novelty of our research in the abstract (lines 11-13), introduction (lines 61-64), and discussion (lines 248-249).

**References**

1. Lawal FB, Dosumu EB. Self-reported and clinically evident gingival bleeding and impact on oral health-related quality of life in young adolescents: a comparative study. Malawi Med J. 2021; 33(2): 121-126.
2. Molina GF, Faulks D, Mazzola I, et al. Three-year survival of ART high-viscosity glass-ionomer and resin composite restorations in people with disability. Clin Oral Investig. 2018; 22(1): 461-467.
3. Chen H, Zhang R, Cheng R, et al. Gingival bleeding and calculus among 12-year-old Chinese adolescents: a multilevel analysis. BMC Oral Health. 2020; 20(1): 147.
4. Loch C, Kuan IB, Elsalem L, et al. COVID‐19 and dental clinical practice: Students and clinical staff perceptions of health risks and educational impact. J Dent Educ. 2021; 85(1): 44-52.
5. Haridy R, Abdalla MA, Kaisarly D, et al. A cross‐sectional multicenter survey on the future of dental education in the era of COVID‐19: Alternatives and implications. J Dent Educ. 2021; 85(4): 483-493.
6. Iyer P, Aziz K, Ojcius DM. Impact of COVID‐19 on dental education in the United States. J Dent Educ. 2020; 84(6): 718-722.
7. Hassona Y, Taimeh D, Marahleh A, et al. YouTube as a source of information on mouth (oral) cancer. Oral Dis. 2016; 22(3): 202-208.
8. Fortuna G, Schiavo JH, Aria M, et al. The usefulness of YouTube™ videos as a source of information on burning mouth syndrome. J Oral Rehabil. 2019; 46(7): 657-665.
9. Nason K, Donnelly A, Duncan HF. YouTube as a patient-information source for root canal treatment. Int Endod J. 2016; 49(12): 1194-1200.
10. Lena Y, Dindaroğlu F. Lingual orthodontic treatment: A YouTube™ video analysis. Angle Orthod. 2018; 88(2): 208-214.
11. Kaval ME, Demirci GK, Atesci AA, et al. YouTube™ as an information source for regenerative endodontic treatment procedures: Quality and content analysis. Int J Med Inform. 2022; 161: 104732.

**Revised text**:

(2) Concern of the reviewer: the methodology seems vague particularly study design? Clarify it.

**Our response**: We are sorry that our research method, especially the study design, was not well explained in the previous response letter. Our study was aimed at a cross-sectional study of YouTube videos related to bleeding gums, as the initial portrayal of the study design section may potentially perplex readers, in light of this, we have made appropriate revisions (lines 68-71), referring to the previous study conducted by Antonio et al [1-3]. Our research design is as follows (The detailed research methods are included in the Materials and Methods section of our manuscript) :

1. Conducted a search on YouTube using the keyword "bleeding gums" from Google Trends, and collected samples using inclusion and exclusion criteria;

2. The descriptive statistics for the videos included the time since upload, the video length, and the number of likes, views, comments, subscribers, and viewing rates;

3. The global quality score (GQS), usefulness score, and DISCERN were used to evaluate the video quality;

4. Statistical analysis was performed using the Kruskal–Wallis test, Mann–Whitney test, and Spearman correlation analysis.

**References**

1. Romano A, Lauritano D, Fiori F, et al. Cross-sectional study on the quality of oral lichen planus videos on YouTube™. J Oral Pathol Med. 2021; 50(2): 220-228.
2. Sezici YL, Gediz M, Dindaroğlu F. Is YouTube an adequate patient resource about orthodontic retention? A cross-sectional analysis of content and quality. Am J Orthod Dentofacial Orthop. 2022; 161(1): e72-e79.
3. Uzel İ, Ghabchi B, Akalın A, et al. YouTube as an information source in paediatric dentistry education: Reliability and quality analysis. PLoS One. 2023; 18(3): e0283300.

**Revised text**:

(3). Concern of the reviewer: the inclusion and exclusion criteria also needs to be clarified.

**Our response**: We are sorry that our previous response letter did not clearly explain the inclusion and exclusion criteria of our study, and the updated explanation is as follows:

1. Because the results may be heavily influenced by inclusion and exclusion criteria, therefore, our study referred to research on information quality analysis of YouTube videos on oral related topics [1-4], which will undoubtedly make the conclusions more solid and reliable. More details on inclusion and exclusion criteria can be found in the manuscript Methods section (lines 97-106).

2. Based on the inclusion and exclusion criteria, we excluded 93 videos. A total of 107 videos were included in further assessments. We mentioned this in the Results section (lines 169-171).

3. We have included comprehensive information about 107 included videos in the S1 Table, and we have also supplemented the exclusion reasons for 93 excluded videos in the S2 Table.

**References**

1. Maganur PC, Hakami Z, Raghunath RG, et al. Reliability of Educational Content Videos in YouTubeTM about Stainless Steel Crowns. Children (Basel). 2022; 9(4): 571.
2. Nason K, Donnelly A, Duncan HF. YouTube as a patient-information source for root canal treatment. Int Endod J. 2016; 49(12): 1194-1200.
3. Sezici YL, Gediz M, Dindaroğlu F. Is YouTube an adequate patient resource about orthodontic retention? A cross-sectional analysis of content and quality. Am J Orthod Dentofacial Orthop. 2022; 161(1): e72-e79.
4. Topsakal KG, Duran GS, Görgülü S, et al. Is YouTubeTM an adequate source of oral hygiene education for orthodontic patients? Int J Dent Hyg. 2022; 20(3): 504-511.

**Revised text**:

(4) Concern of the reviewer: How you accessed the video quality? is there any gold standard?

**Our response**: We are sorry that we did not explain clearly how we accessed the video quality in our previous response letter. As there is still no gold standard for evaluating video quality, therefore, our study referred to several recent studies on information quality analysis of YouTube videos on oral related topics using the global quality score (GQS) [1,2] and usefulness score [3] to evaluate video quality. While video reliability was assessed using DISCERN [4] (A full explanation of the methods used to access video quality analysis can be found in the manuscript Methods section (lines 121-148)). While these are mature standards, we look forward to a gold standard to be developed for evaluating video quality in the future that will help improve video quality by monitoring health information on YouTube and other social networks.

**References**

1. Sezici YL, Gediz M, Dindaroğlu F. Is YouTube an adequate patient resource about orthodontic retention? A cross-sectional analysis of content and quality. Am J Orthod Dentofacial Orthop. 2022; 161(1): e72-e79.
2. Ustdal G, Guney AU. YouTube as a source of information about orthodontic clear aligners. Angle Orthod. 2020; 90(3): 419-424.
3. Çapan BŞ. YouTube as a source of information on space maintainers for parents and patients. PLoS One. 2021; 16(2): e0246431.
4. Charnock D, Shepperd S, Needham G, et al. DISCERN: an instrument for judging the quality of written consumer health information on treatment choices. J Epidemiol Community Health. 1999; 53(2): 105-111.

**Revised text**: not applicable.

**Reviewer 3:**

(1)introduction line 29- its not explanatory, patients frequently present to the dental clinic with bleeding gums while brushing their teeth or noticing blood in their mouth in the morning after waking up.

**Our response**: We would like to express our gratitude to the reviewer for pointing out this error, as it will enhance the precision of the sentence. We have revised it (introduction, lines 29-31) , and have appropriately cited the corresponding literature in the reference list (References 1).

**Revised text**:

(2) line 33- classification is 2017 not 2018.

**Our response**: We thank the reviewer for pointing out this error and we have revised the year for the new classification of periodontal and peri-implant diseases and conditions (introduction, lines 35), which was proposed in 2017 as said by the reviewer [1].

**References**

1. Caton JG, Armitage G, Berglundh T, Chapple IL, Jepsen S, Kornman KS, et al. A new classification scheme for periodontal and peri‐implant diseases and conditions–Introduction and key changes from the 1999 classification. Journal of clinical periodontology. 2018; 45 Suppl 20: S1-S8.

**Revised text**:

(3) what were your keywords for search?

**Our response**: We are grateful to the reviewer for bringing this matter to our attention, referring to the information quality analysis study of YouTube videos on oral related topics [1-3], we were performed on YouTube using the keyword "bleeding gums" from Google Trends (details about the search strategies are included in the Materials and Methods section of our article (lines 73-84)).

**References**

1. Maganur PC, Hakami Z, Raghunath RG, Vundavalli S, Jeevanandan G, Almugla YM, et al. Reliability of Educational Content Videos in YouTubeTM about Stainless Steel Crowns. Children (Basel). 2022; 9(4): 571.
2. Sezici YL, Gediz M, Dindaroğlu F. Is YouTube an adequate patient resource about orthodontic retention? A cross-sectional analysis of content and quality. Am J Orthod Dentofacial Orthop. 2022; 161(1): e72-e79.
3. Topsakal KG, Duran GS, Görgülü S, et al. Is YouTubeTM an adequate source of oral hygiene education for orthodontic patients? Int J Dent Hyg. 2022; 20(3): 504-511.

**Revised text**: not applicable.

Thank you for your consideration. I look forward to hearing from you.

Best regards,

Prof. Minkui Lin

(Fujian Key Laboratory of Oral Diseases&Fujian Provincial Engineering Research Center of Oral Biomaterial&Stomatological Key Lab of Fujian College and University, School and Hospital of Stomatology, Fujian Medical University)

Email address: linmk105@ sina.com
