# Supplementary material for: YouTube as an information source for bleeding gums: A quantitative and qualitative analysis
Source: PLoS One. 2024 Mar 6;19(3):e0298597. doi: 10.1371/journal.pone.0298597 (PMC10917331; doi:10.1371/journal.pone.0298597)
Supplement: Attachment — Submitted filename: Response to Reviewers.docx [file pone.0298597.s005.docx]

Dear Editor:

Thank you for giving us the opportunity to submit a revised version of “YouTube as a Source of Information on Bleeding Gums: A Quantitative and Qualitative Analysis” (PLOS ONE manuscript number: PONE-D-23-16369) for potential publication in PLOS ONE. We appreciate your and the reviewers’ time and effort in providing feedback on our article. Your views, as well as the reviewers’, are valuable and have been instrumental in improving our research papers. We have carefully reviewed all of the suggestions and have made our best efforts to incorporate them into the text in order to meet the acceptance criteria. The following is our peer-to-peer response to these comments:

**Reviewer Comments and Responses:**

1. If the authors have adequately addressed your comments raised in a previous round of review and you feel that this manuscript is now acceptable for publication, you may indicate that here to bypass the “Comments to the Author” section, enter your conflict of interest statement in the “Confidential to Editor” section, and submit your "Accept" recommendation.

Reviewer #1: All comments have been addressed

Reviewer #3: All comments have been addressed

**Our response**: We express our gratitude to the reviewers for their endorsement. .

2. Is the manuscript technically sound, and do the data support the conclusions?

Reviewer #1: Yes

Reviewer #3: Yes

**Our response**: None.

3. Has the statistical analysis been performed appropriately and rigorously?

Reviewer #1: Yes

Reviewer #3: Yes

**Our response**: None.

4. Have the authors made all data underlying the findings in their manuscript fully available?

Reviewer #1: Yes

Reviewer #3: Yes

**Our response**: None.

5. Is the manuscript presented in an intelligible fashion and written in standard English?

Reviewer #1: Yes

Reviewer #3: Yes

**Our response**: None.

6. Review Comments to the Author

**Reviewer #1:** Dear Author,

Rebuttal against the queries are not addressed satisfactorily. The manuscript is not acceptable in current form.

We appreciate Dr. Akhilanand Chaurasia’s acknowledgement of our efforts to improve the manuscript based on the comments in the first round of review. However, there are still several areas that require further clarification. We have tried our best to review the manuscript and made sure that the updated responses below fully meet your requirements.

(1) What is novelty of your research ?

**Our response**: According to our knowledge, no recent study has evaluated content related to bleeding gums on YouTube. However, study on YouTube video relevant to bleeding gums is required, as bleeding gums present a prevalent dental issue in individuals' everyday lives [1-3]. Bleeding gums are mainly triggered by periodontal disease and occasionally by peri-implant diseases, direct trauma, viruses, fungal or bacterial infections, medications, pregnancy, dermatoses, and systemic disorders [4-12], patients' quality of life suffers as a result [13-15].

The utilization of the YouTube platform for the purpose of seeking health-related information has become prevalent among individuals [16], and it is important to note that YouTube is open access, which means that user-generated content is not peer reviewed; while it offers effective health education resources, there may also be a great deal of misinformation and inaccuracy [17]. Incorrect and inadequate content in YouTube videos about bleeding gums can significantly influence patients' attitudes and medical decisions. Consequently, the objective of this study is to perform both quantitative and qualitative analyses of YouTube videos pertaining to bleeding gums.

In our article, we have directly stated the novelty of our research in the abstract (lines 11-13), introduction (lines 61-64), and discussion (lines 248-249). In addition, we added relevant references to the discussion, highlighting studies with both intra-oral and extra-oral causes, such as bad breath [18], with similar results [19], reinforcing our conclusions. At the same time, in order to better evaluate YouTube videos, we also refer to the literature of Chaurasia et al. [20], and add the possible role of Deep Learning model related content in YouTube video evaluation. Although it has not been realized in our study, Deep Learning model provides an important direction for future application.

**References**

1. Lawal FB, Dosumu EB. Self-reported and clinically evident gingival bleeding and impact on oral health-related quality of life in young adolescents: a comparative study. Malawi Med J. 2021; 33(2): 121-126.
2. Molina GF, Faulks D, Mazzola I, et al. Three-year survival of ART high-viscosity glass-ionomer and resin composite restorations in people with disability. Clin Oral Investig. 2018; 22(1): 461-467.
3. Chen H, Zhang R, Cheng R, et al. Gingival bleeding and calculus among 12-year-old Chinese adolescents: a multilevel analysis. BMC Oral Health. 2020; 20(1): 147.
4. Newbrun E. Indices to measure gingival bleeding. Journal of periodontology. 1996; 67(6): 555-561.
5. Darby I. Drugs and gingival bleeding. Australian Prescriber. 2006; 29: 154-155.
6. Bui FQ, Almeida-da-Silva CLC, Huynh B, Trinh A, Liu J, Woodward J, et al. Association between periodontal pathogens and systemic disease. Biomedical journal. 2019; 42(1): 27-35.
7. Rokaya D, Srimaneepong V, Wisitrasameewon W, Humagain M, Thunyakitpisal P. Peri-implantitis Update: Risk Indicators, Diagnosis, and Treatment. European journal of dentistry. 2020; 14(4): 672-682.
8. Heboyan A, Syed AUY, Rokaya D, Cooper PR, Manrikyan M, Markaryan M. Cytomorphometric Analysis of Inflammation Dynamics in the Periodontium Following the Use of Fixed Dental Prostheses. Molecules. 2020; 25(20): 4650.
9. Elani HW, Starr JR, Da Silva JD, Gallucci GO. Trends in Dental Implant Use in the U.S., 1999-2016, and Projections to 2026. Journal of dental research. 2018; 97(13): 1424-1430.
10. Lee CT, Huang YW, Zhu L, Weltman R. Prevalences of peri-implantitis and peri-implant mucositis: systematic review and meta-analysis. Journal of dentistry. 2017; 62: 1-12.
11. Achoki T, Miller-Petrie MK, Glenn SD, Kalra N, Lesego A, Gathecha GK, et al. Health disparities across the counties of Kenya and implications for policy makers, 1990-2016: a systematic analysis for the Global Burden of Disease Study 2016. Lancet Glob Health. 2019; 7(1): e81-e95.
12. Pihlstrom BL, Michalowicz BS, Johnson NW. Periodontal diseases. Lancet. 2005;366(9499):1809-1820.
13. Santonocito S, Palazzo G, Indelicato F, Chaurasia A, Isola G. Effects induced by periodontal disease on overall quality of life and self-esteem. Mediterranean Journal of Clinical Psychology. 2022; 10(1).
14. Slots J. Periodontology: past, present, perspectives. Periodontol 2000. 2013;62(1):7–19.
15. Frencken JE, Sharma P, Stenhouse L, Green D, Laverty D, Dietrich T. Global epidemiology of dental caries and severe periodontitis—a comprehensive review. Journal Of Clinical Periodontology. 2017;44(Suppl 18):S94-s105.
16. Bezner SK, Hodgman EI, Diesen DL, Clayton JT, Minkes RK, Langer JC, et al. Pediatric surgery on YouTube™: is the truth out there? Journal of Pediatric Surgery. 2014; 49(4): 586-589.
17. Hassona Y, Taimeh D, Marahleh A, Scully C. YouTube as a source of information on mouth (oral) cancer. Oral Dis. 2016; 22(3): 202-208.
18. Chaurasia A, Katheriya G. Prevalence of halitosis and related factors in North Indian population- A hospital based cross-sectional study. Journal of Oral Medicine, Oral Surgery, Oral Pathology and Oral Radiology. 2018; 4(2):79-84.
19. Ramadhani A, Zettira Z, Rachmawati YL, Hariyani N, Maharani DA. Quality and Reliability of Halitosis Videos on YouTube as a Source of Information. Dentistry Journal (Basel). 2021; 9(10): 120.
20. Chaurasia A, Namachivayam A, Koca-Ünsal RB, Lee JH. Deep-learning performance in identifying and classifying dental implant systems from dental imaging: a systematic review and meta-analysis. Journal of Periodontal and Implant Science. 2023; 53(3):e12.

**Revised text**:

(2) Concern of the reviewer: the methodology seems vague particularly study design? Clarify it.

**Our response**: We appreciate the opportunity to explain our research method (especially the study design). Our research design is a cross-sectional study that quantitatively and qualitatively analyzes videos related to bleeding gums on YouTube. Within the framework of this research design, referring to the previous study conducted by Antonio et al [1-3], we provide a detailed description of the specific techniques and procedures used to collect, analyze, and interpret data in the Materials and Methods section. Our research method is as follows (The detailed research methods are included in the Materials and Methods section of our manuscript):

1. Conducted a search on YouTube using the keyword "bleeding gums" from Google Trends, select the top 200 videos, and further analyze 107 of them based on the inclusion and exclusion criteria;

2. Descriptive statistics on 107 videos, including the time since upload, the video length, and the number of likes, views, comments, subscribers, and viewing rates, and categorize these videos according to their sources;
3. Use global quality score (GQS), usefulness score, and total score to evaluate video quality, and use DISCERN to evaluate video reliability;
4. IBM SPSS Statistics 27 software was used for statistical tests. The normality of the data was determined using the Shapiro‒Wilk test. Non-parametric tests were used for data that did not adhere to the normal distribution: the Kruskal-Wallis test (for more than two samples), the Mann-Whitney test (for two independent samples), and Spearman's correlation coefficient. Kappa coefficients of correlation were calculated to assess interrater reliability. The significance level was set at p <0.05.

**References**

1. Romano A, Lauritano D, Fiori F, et al. Cross-sectional study on the quality of oral lichen planus videos on YouTube™. J Oral Pathol Med. 2021; 50(2): 220-228.
2. Sezici YL, Gediz M, Dindaroğlu F. Is YouTube an adequate patient resource about orthodontic retention? A cross-sectional analysis of content and quality. Am J Orthod Dentofacial Orthop. 2022; 161(1): e72-e79.
3. Uzel İ, Ghabchi B, Akalın A, et al. YouTube as an information source in paediatric dentistry education: Reliability and quality analysis. PLoS One. 2023; 18(3): e0283300.

(3) Concern of the reviewer: the inclusion and exclusion criteria also needs to be clarified.

**Our response**: We are sorry that our previous response letter did not clearly explain the inclusion and exclusion criteria of our study, and the updated explanation is as follows:

1.Since the results may be heavily influenced by the inclusion and exclusion criteria, we referred to research on information quality analysis of YouTube videos on oral related topics [1-4] when formulating the inclusion and exclusion criteria, aiming to select research objects with common characteristics from complex videos and exclude the influence of some non-research factors. Ensuring the reasonableness and completeness of the inclusion and exclusion criteria will undoubtedly make the conclusions more solid and reliable. Detailed information on the inclusion and exclusion criteria can be found in the manuscript Methods section (lines 97-106).

2. Based on the inclusion and exclusion criteria, we excluded 93 videos. A total of 107 videos were included in further assessments. We mentioned this in the Results section (lines 169-171).

3. We have included comprehensive information about 107 included videos in the S1 Table, and we have also supplemented the exclusion reasons for 93 excluded videos in the S2 Table.

**References**

1. Maganur PC, Hakami Z, Raghunath RG, et al. Reliability of Educational Content Videos in YouTubeTM about Stainless Steel Crowns. Children (Basel). 2022; 9(4): 571.
2. Nason K, Donnelly A, Duncan HF. YouTube as a patient-information source for root canal treatment. Int Endod J. 2016; 49(12): 1194-1200.
3. Sezici YL, Gediz M, Dindaroğlu F. Is YouTube an adequate patient resource about orthodontic retention? A cross-sectional analysis of content and quality. Am J Orthod Dentofacial Orthop. 2022; 161(1): e72-e79.
4. Topsakal KG, Duran GS, Görgülü S, et al. Is YouTubeTM an adequate source of oral hygiene education for orthodontic patients? Int J Dent Hyg. 2022; 20(3): 504-511.

(4) Concern of the reviewer: How you accessed the video quality? is there any gold standard?

**Our response**: We are sorry that we did not explain clearly how we accessed the video quality in our previous response letter. As there is still no gold standard for evaluating video quality, therefore, our study referred to several recent studies on information quality analysis of YouTube videos on oral related topics. Global quality score (GQS) [1,2] and usefulness score [3] were respectively used to evaluate the quality of YouTube video information to improve the accuracy of quality analysis results. The results showed that the quality scores of videos using GQS and usefulness scores were consistent, respectively (A full explanation of the methods used to access video quality analysis can be found in the manuscript Methods section (lines 121-148)). While these are mature standards, we look forward to a gold standard to be developed for evaluating video quality in the future that will help improve video quality by monitoring health information on YouTube and other social networks.

**References**

1. Sezici YL, Gediz M, Dindaroğlu F. Is YouTube an adequate patient resource about orthodontic retention? A cross-sectional analysis of content and quality. Am J Orthod Dentofacial Orthop. 2022; 161(1): e72-e79.
2. Ustdal G, Guney AU. YouTube as a source of information about orthodontic clear aligners. Angle Orthod. 2020; 90(3): 419-424.
3. Çapan BŞ. YouTube as a source of information on space maintainers for parents and patients. PLoS One. 2021; 16(2): e0246431.

**Reviewer #3:**

All the queries and references are well explained and addressed to in proper systematic manner. all required questions have been answered and that all responses meet formatting specifications.

**Our response**: We thank Reviewer #3 for recognition of our revised manuscript.

Thank you for your consideration. I look forward to hearing from you.

Best regards,

Prof. Minkui Lin

(Fujian Key Laboratory of Oral Diseases&Fujian Provincial Engineering Research Center of Oral Biomaterial&Stomatological Key Lab of Fujian College and University, School and Hospital of Stomatology, Fujian Medical University)

Email address: linmk105@ sina.com
